# Complete mitochondrial genome of the radicine pond snail *Radix plicatula* (Gastropoda: Lymnaeidae)

**DOI:** 10.1080/23802359.2019.1661300

**Published:** 2019-09-06

**Authors:** Dong-Mei Qin, Xiao-Chen Huang, Li-Min Yang, Xiong-Jun Liu, Rui-Wen Wu, Shan Ouyang, Xiao-Ping Wu, Shang-Hong Wang

**Affiliations:** a School of Life Sciences, Nanchang University, Nanchang, People’s Republic of China;; b Poyang Lake Key Laboratory of Environment and Resource Utilization, Ministry of Education, Nanchang University, Nanchang, People’s Republic of China;; c School of Resource, Environment and Chemical Engineering, Nanchang University, Nanchang, People’s Republic of China

**Keywords:** Amphipepleinae, freshwater snail, mitochondrial genome, phylogeny, *Radix plicatula*

## Abstract

*Radix plicatula* is broadly distributed in China, as well as Russia. It is one of the intermediate hosts of *Fasciola* species which leads to the spread of fascioliasis. Here, we first described the complete mitochondrial genome of *R. plicatula*. The mitogenome is 13,751 bp in length, containing 13 protein-coding genes, 22 tRNA genes, and 2 rRNA genes. The contents of each base are 30.7% A, 39.6% T, 15.7% G, and 13.9% C. The sequence is AT rich (70.3%). Mitochondrial phylogenomic analysis showed that *R. plicatula* is close to *R. auricularia*.

*Radix plicatula* is a species of aquatic pulmonate gastropod mollusk in the family Lymnaeidae, broadly distributed in China, as well as in Russia (Liu et al. 1993). *Radix* species is one of the intermediate hosts of *Fasciola* species, which play a critical role in the spread of fascioliasis in humans (Correa et al. 2010; Lawton et al. 2015). Currently, approximately 20 million people have been infected with fascioliasis worldwide (Correa et al. 2010). Due to the high plasticity of the shell shape within the *Radix* species, it is difficult to classify them only according to morphological features (Pfenninger et al. 2006; Feldmeyer et al. 2010). Recently, some studies have performed the classification and phylogenetic analysis of *Radix* species and its allies (Bargues et al. 2001; Lawton et al. 2015; Aksenova et al. 2018). So far, however, in *Radix* only one species (i.e. *R. auricularia* from Germany) had the complete mitochondrial genome.

Here, we sequenced and annotated the complete mitochondrial genome of *R. plicatula* and reconstructed the phylogenetic relationships of 12 gastropod molluscs with the focus on *Radix sensulato*. Our results provide new insights into the relationship of radicine pond snails and genetic information for controlling the spread of Fascioliasis.

Specimens were collected from ponds in Nanchang (28°39′39″N, 115°47′20″E), China, in 2019. Morphological identification was following the literature (Yen 1939; Liu et al. 1993). The voucher specimens were deposited in the Nanchang University, Nanchang, China. Foot tissue of each specimen was preserved in −80 °C and then one individual (voucher number: ncuwxp-nc-201901) was used for genomic DNA extraction, followed by the Illumina Miseq sequencing platform with the strategy of 2 × 250 bp paired-ends. Complete mitochondrial genome was generated according to the previous study (Zhou et al. 2019).

Complete mtDNA sequence of *R. plicatula* is 13,751 bp in length (GenBank accession number: MN175602) and contains 13 protein-coding genes (PCGs), 22 tRNA genes, and 2 rRNA genes. The light strand includes 11 genes (four PCGs, *rrnS* gene, and six tRNA genes) and the heavy strand includes 26 genes (nine PCGs, *rrnL* gene, 16 tRNA genes). The overall base composition of the mitochondrial genome is as follows: 30.7% A, 39.6% T, 15.7% G, and 13.9% C. The sequence is AT-rich (70.3%), similar to other Panpulmonata species (Feldmeyer et al. 2010; Liu et al. 2012; Zhou et al. 2019).

Compared with *R. auricularia* mitogenome, the gene order of *R. plicatula* is identical, but different from that of *Ampullaceana balthica* because of the positional mismatch for the tRNA-Trp gene. Phylogenetic analysis based on 12 PCGs and 2 rRNA genes showed that the family Lymnaeidae is monophyletic (BS = 100), and *R. plicatula* and *R. auricularia* had a close relationship (BS = 100) (Figure 1).

**Figure 1.**
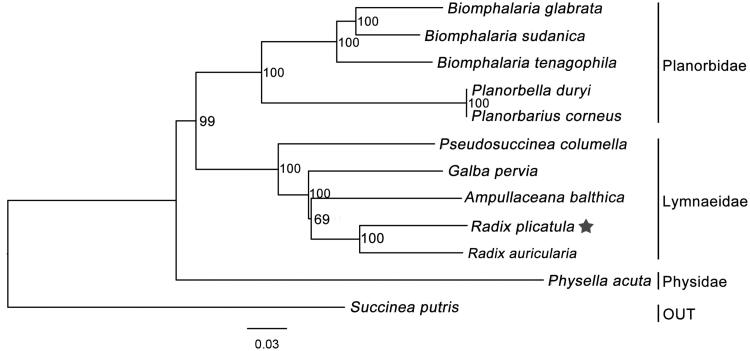
Phylogenetic relationships of 12 gastropod molluscs using neighbour-Joining method. (GenBank accession numbers, *Ampullaceana balthica*: NC_026539; *Radix plicatula*: MN175602; *Galba pervia*: NC_018536; *Radix auricularia*: NC_026538; *Pseudosuccinea columella*: MH614274; *Planorbarius corneus*: NC_026708; *Biomphalaria tenagophila*: NC_010220; *Planorbella duryi*: KY514384; *Biomphalaria sudanica*: NC_038060; *Biomphalaria glabrata*: NC_005439; *Physella acuta*: NC_023253; *Succinea putris*: NC_016190).
